# Revisiting the Effect of U-Bends, Flow Parameters, and Feasibility for Scale-Up on Residence Time Distribution Curves for a Continuous Bioprocessing Oscillatory Baffled Flow Reactor

**DOI:** 10.1021/acs.iecr.2c00822

**Published:** 2022-07-19

**Authors:** Rylan Cox, Konstantinos Salonitis, Evgeny Rebrov, Susan A. Impey

**Affiliations:** † School of Aerospace, Transport and Manufacturing, Cranfield University, Cranfield MK43 0AL, U.K.; ‡ School of Engineering, University of Warwick, Coventry CV4 7AL, U.K.; § Department of Chemical Engineering and Chemistry, Eindhoven University of Technology, P.O. Box 513, 5600 MB Eindhoven, The Netherlands

## Abstract

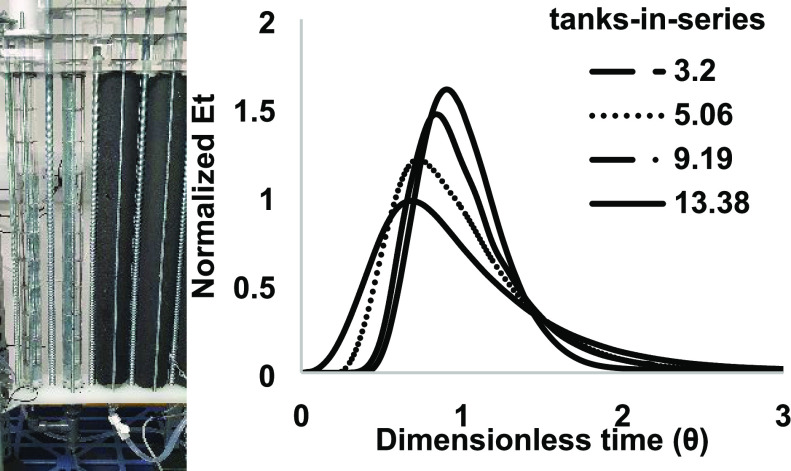

An oscillatory baffled flow reactor (OBR) has been designed with 60 interbaffled cells. The baffled columns of 40 mm internal diameter together result in a reactor length of 5740 mm. The oscillatory amplitude and frequency were in the range of 2–12 mm and 0.3–2 Hz, respectively. The report investigates the impact of U-bends and the number of reactor sections on axial dispersion for scale-up feasibility. A prediction model using operating parameters has been developed to maximize plug flow conditions using the tanks-in-series (TiS) model. The maximum TiS value was 13.38 in a single column compared to 43.68 in the full reactor at a velocity ratio of 2.27 using oscillatory parameters 8 mm and 0.3 Hz. The mixing efficiency along the reactor was found to decrease after each column at amplitudes <6 mm compared to amplitudes up to 12 mm, where a negligible impact was observed. U-bend geometry had a significant role in the decrease of TiS values.

## 1 Introduction

Bioprocessing is a fast-paced thriving sector that has many different industrial spokes with a wide variety of products, which include the conversion of biomass into fuels,^1,2^ production of raw materials otherwise produced from chemical processes,^3,4^ production of secondary metabolites,^5^ wastewater treatment,^6^ enzyme production,^7^ and vaccine and active pharmaceutical ingredient (API) drug manufacture.^8^ Traditionally, a significant number of bioprocesses are conducted in large vats in a batch process or adopted for continuous processes.^9^ Conventional technology such as stirred tank reactors (STRs) or continuous stirred tank reactors (CSTRs) suffer from inadequate mixing conditions,^10^ reduced production rates, and limited mass transfer, particularly that of oxygen, which is already a rate-limiting factor in aerobic fermentation processes.^11^

On the other hand, technologies such as the oscillatory baffled flow reactor (OBR) aim to shift industrial biotechnology toward continuous manufacturing. One biotechnology company currently operates a commercial OBR for their production of enzymes, but due to confidentiality, they have not disclosed the reasons for using the OBR.^7^ Other laboratory-scale demonstrations that have successfully enhanced the production rate or performance include crystallization,^12^ fermentation processes,^2^ and production of biofuel,^13^ polymers,^10^ and microalgae.^14^ Continuous flow technologies are appealing to the bioprocessing industry due to the potential in reducing footprint, waste, cost, and energy compared to the batch process^15^ but are yet to reach industrial expectations. OBRs are suitable for bioprocesses due to their increased production per unit volume,^9^ low shear rates reducing stress on cells,^16^ uniform and controllable mixing independent of net flow allowing controllable residence time,^17^ and improved scale-up capabilities.^12^

OBR technology relies on periodically spaced baffles within columns, in which oscillation is applied either by reciprocating baffles^10^ or by fluid pulsations.^18^ As the fluid oscillates back and forth against the constrictions, eddies are formed on either side of the baffle. This results in the absence of dead zones or points where no mixing occurs, achieving plug flow characteristics and uniform mixing by altering oscillation parameters.^19^ When OBRs are operated continuously, the application of oscillation within the system decouples the mixing from the net flow, whereby a pump drives the fluid forward, while the oscillatory motion controls the mixing conditions.^20^ Similar to conventional tubular reactors, where areas of no mixing are found close to the walls, CSTRs suffer from areas of no mixing due to propeller inefficiencies.

Scaling up OBR technology while maintaining near-plug-flow conditions is controlled through dimensionless numbers: Strouhal, Reynolds number, and oscillatory Reynolds number.^21^ CSTR mixing efficiency reduces with scale-up, whereas OBR scale-up is said to be linear and therefore becomes more appealing for production plants.^22^ Current research indicates that near-plug flow is achieved at a velocity ratio between 4 and 8,^22^ which is the ratio between the net flow and the oscillatory parameters described later. Sutherland et al. reported a more suitable velocity ratio between 2.5 and 3.5;^23^ however, these ranges may not be optimal with varied OBR geometries such as baffle sizing or spacing, U-bend inclusions, or scaling up.

Conventional tubular reactor systems for bioprocessing would require an impractical reactor length to match the long residence times. OBRs can be adopted for continuous manufacturing bioprocesses with long residence times through two routes: first, by expanding tube diameters and maintaining the proven single or multiorifice baffled design;^24,25^ second, by extending the reactor length with longer or more baffled columns connected with U-bends.^19^ Additional U-bends and columns increase pressure drop across the reactor from increased friction and momentum changes, potentially dampening oscillations. Furthermore, bioprocessing often requires the addition of gas sparging of oxygen or carbon dioxide. Identifying the influence of fluid oscillation and plug flow characteristics when scaling up through these routes, as well as the impact from sparging points and gas vents, requires further investigation on their impact on residence time distribution (RTD) curves and tanks-in-series (TiS) number.^16^

Experimental design methodologies are often used to quantify the axial dispersion within OBRs through the use of a TiS model.^26^ Design of experiments (DOE) to evaluate key operational parameters such as amplitude (*x*_0_), frequency (*f*), and their corresponding dimensionless numbers is used to maximize plug flow within the OBR system.^9,19,21,27,28^ Other studies have investigated the impact of oscillatory parameters, tube diameter, geometric designs, and baffle types on both axial dispersion and mass transfer when scaling OBRs but usually have confined it to a single column identified in Table 1. Sutherland et al. expected mixing time to decrease with tube diameter scale-up; however, when numerically modeled, a proportional increase in mixing time was observed using geometric similarity in a moving baffled column when scaling.^29^ Ahmed et al. found that when scaling a helical baffled column at three different scales by extending the column length and tube diameter, RTD curves are not affected, provided there is a geometric similarity and dimensionless numbers remain the same across scales. In their study, a model was also developed to predict the TiS value as a function of dimensionless numbers when scaling a single-column helical baffled system.^30^ The same author also investigated the scale-up of a single-columned multiorifice oscillatory baffled reactor for gas–liquid mass transfer by increasing the tube diameter and column height. Ahmed et al. found that only the slug flow region could be produced in the mesoscale OBR under the tested parameters. Furthermore, the mass transfer coefficient increased with an increase in the tube diameter.^31^ Ni et al. studied different single-orifice baffles when scaling with the tube diameter using the axial dispersion model in a single column both experimentally and with simulated analysis. For the conventional close-fit single-orifice OBR design, experimental and simulated data suggested that the axial dispersion coefficient scales linearly when increasing the tube diameter for both batch and continuous systems.^24,32^ Smith et al. developed three OBRs at different scales with geometric similarity and found that the axial dispersion was not a function of tube diameter when scaling through this route.^25^

**Table 1:** Scale-Up Studies Conducted on OBRs Consisting of Different Geometries, Scale-Up Strategies, and Analysis

studies	investigation	simulation or experimental study	scale-up type	baffle type	column number	reference
1	residence time distribution	simulation	tube diameter	moving single-orifice baffle	single column	(29)
2	residence time distribution	experimental	tube diameter and length	helical baffle	single column	(30)
3	mass transfer	experimental	tube diameter	multiorifice	single column	(31)
4	axial dispersion	experimental	tube diameter and length	single orifice	single and multicolumn	(33)
5	axial dispersion	experimental	tube diameter and length	single orifice	single column	(24)
6	axial vs tangential velocity ratio	simulation	tube diameter	single orifice	single column	(32)
7	design methodology		tube diameter and length	single orifice	multicolumn	(19)
8	axial dispersion	experimental	reactor length	single orifice	multicolumn	(25)
9	tanks-in-series	experimental	reactor length, U-bend effect, and tube diameter	single orifice	multicolumn	this work

Nevertheless, investigation of the scale-up feasibility for continuous OBR systems is not as well documented regarding the reactor length with multiple columns for both axial dispersion and OBR mixing efficiency, which may become limited due to the dampening of oscillations.^12,25^ Stonestreet and Harvey identified a design methodology for scaling up OBRs based on process residence times and required throughput. It was identified that OBRs should be scaled by the reactor length and increasing tube diameter while maintaining geometric similarity to mesoscale OBRs and dimensionless operating parameters such as oscillatory Reynolds number, Strouhal number, and velocity ratio. However, this study was under the assumption of uniform oscillations along the entire reactor length due to a pressurized double oscillatory piston working antiphase to each other at the inlet and outlet; when in reality, it is likely that the midpoint of the reactor length exhibited little to no oscillation in OBRs hundreds of meters in length.^19^ Ni and Pereira investigated a 14-column OBR, each 1000 mm in length, with connecting U-bends using both the plug flow with axial dispersion model and a continuous stirred tank with the feedback model. In the study, low values of axial dispersion were achieved with Gaussian distribution RTD curves produced along the reactor length; however, no exact quantitative value was given for axial dispersion at each section across the reactor length.^33^

In this article, the RTD was investigated for a novel design of an OBR operated with various *x*_0_ and *f* values. The RTD was quantified using the TiS model to calculate the number of CSTRs in series. The TiS model was chosen over the axial dispersion model as it has been used extensively in previous studies to characterize the mixing efficiency in OBRs,^9,22,34−36^ its simplicity and robustness,^7^ and independence from adding or removing different sections within a reactor, making the addition of columns possible without impacting the model, as well as the independence to reactor length and flow rate.^26^ The TiS model with back-mixing was not used, as the oscillatory parameters did not exceed 16 mm (0.23 times the baffle length) or *f* > 20 Hz as stated by Avila et al.^7^ Application of a deconvolution through the time domain using fast-Fourier transformation (FFT) was evaluated to find the true RTD profiles.^37−39^ An OBR system of 7 L with a tube diameter of 40 mm was used for this study. A total of five baffled columns were used to evaluate the RTD along the reactor length, noting both the effect of connecting U-bends and reactor length. Considering RTD profiles along the reactor under different oscillatory conditions helps to identify any impact on oscillation dampening when scaling through the reactor length and the effect of connecting parts like U-bends. Obtained TiS values and RTD profiles are compared to those achieved in current research.

## 2 Materials and Methods

### 2.1 Experimental Setup

**Figure 1:**
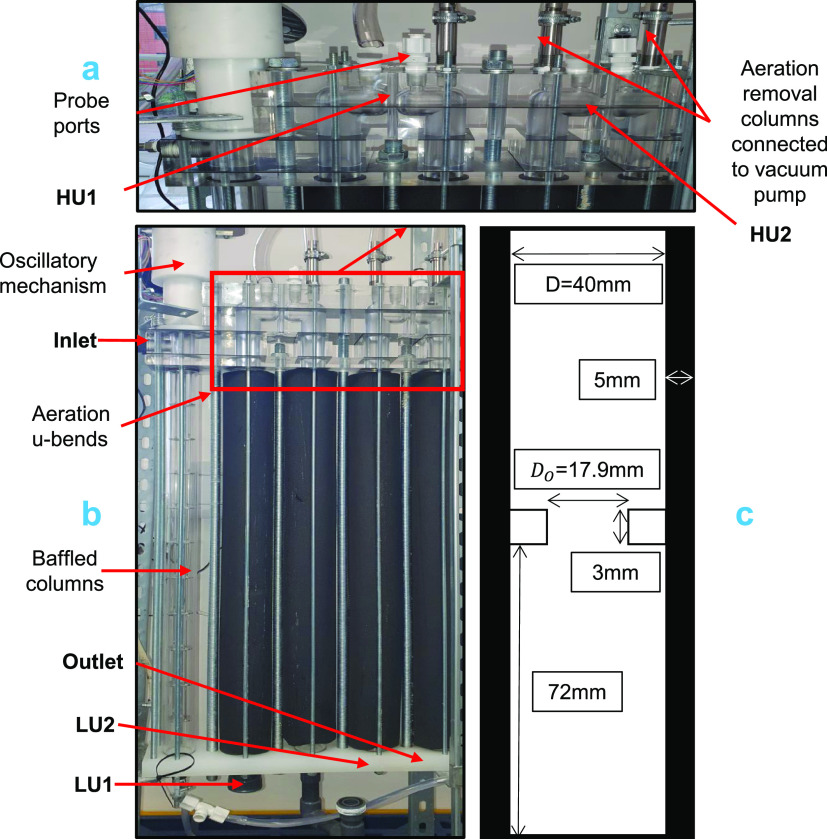
Experimental setup of the oscillatory baffled flow reactor; (a) top U-bend with aeration vents and probe ports; (b) full-scale; (c) baffled column dimensions. Notations LU and HU indicate lower U-bend and higher U-bend with the number ascending based on the direction of flow.

An oscillatory baffled flow reactor (OBR) of 7 L was constructed with five columns in a vertical orientation for continuous operation. Acrylic tubes of 40 mm internal diameter (*D*) and 50 mm outer diameter containing SS316 baffled columns are shown in Figure 1a. SS316 was used for corrosive resistance, as it is an industry standard for many bioprocessing reactors. Each baffled column consisted of 12 interbaffled zones equally spaced at a distance of 1.8 *D*; the value for maximizing mass transfer was the same as in Ni et al. study,^40^ with baffles connected via 3 mm diameter rods, which act as smooth appendages without impacting the flow.^41^ Each baffle was 3 mm in thickness with a constriction ratio (α) of 20% following eq 1, where *D* is the tube diameter and *D*_O_ is the baffle orifice diameter at 17.9 mm, as shown in the schematic in Figure 1c.
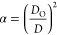
1

Each column is connected by U-bends adopted for gas sparging into the reactor. At the base, poly(vinyl chloride) (PVC) U-bends of 19 mm internal diameter were used, with a T-junction at the base of the riser fitted with a sparging unit to allow aeration. The top two acrylic U-bends had a central baffle added to evaluate plug-flow characteristics with and without a baffle. This baffle had a smooth constriction as opposed to the column’s sharp constriction due to machinability ease. The top U-bends had two probe ports for tracer measurement immediately before and after the baffle. Two SS316 venting columns were fitted along the center point of each OBR column, as shown in Figure 1b. Each SS316 venting column had a 15D07MI oleophobic semipermeable membrane provided by Sartorius, sandwiched within to allow gas to vent out. The superimposed oscillatory flow was produced via a sinusoidal scotch yolk mechanism capable of a frequency of 0.05–2 Hz and an amplitude of 0.5–12 mm. The ranges of frequency and amplitude were carefully selected to ensure a broad range of oscillatory Reynolds numbers with different parameters. The oscillatory Reynolds number dictates the mixing intensity within the OBR following eq 2 through parameters *f* and *x*_O_. The net flow was provided by a peristaltic pump controlled by in-house electronics. The connection tubing to circulate from the reactor inlet and outlet was 12 mm ID polyethylene tubing.

### 2.2 Oscillatory Baffled Flow Reactor Parameters

OBRs are characterized by three dimensionless numbers: oscillatory Reynolds number (*Re*_o_), Reynolds number (*Re*_n_), and Strouhal number (*St*), as shown in eqs 2–4.^19^
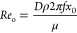
2
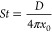
3
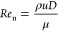
4where ρ is the fluid density (kg m^–3^), μ is the dynamic viscosity (Pa s), and *u* is the linear fluid velocity (m s^–1^). Fluid density is assumed to be constant using water at a value of 1000 kg m^–3^ and a dynamic viscosity of 0.001 Pa s. The tube diameter (*D*) was fixed at 40 mm, and the linear velocity was fixed at 0.007 m s ^–1^, corresponding to a flow rate of 0.0083 L s^–1^ and *Re*_n_ of 263. As the study focused on the effect of oscillatory parameters and scale-up feasibility, a single flow rate was used.

*Re*_o_ describes the intensity of oscillatory mixing within the column, *St* represents the eddy propagation between each interbaffled zone where *St* is inversely proportional to *x*_0_, and finally *Re*_n_ is the Reynolds number that determines the ratio of inertial force with viscous force from the net flow within a tube.^16^*Re*_o_ must be higher than the *Re*_n_ to ensure the eddies produced by oscillatory motion are not carried off downstream before fully evolving.^7^Equation 5 determines the ratio between the two Reynolds numbers for identification if the value is above 1, indicating a degree of flow reversal, and used as a guideline to determine the plug-flow conditions within the reactor.^42^ This ratio is known as the velocity ratio (ψ), where in this study, the range assessed was from 0.28 to 15.92. This range was selected based on current observations in the literature for maximizing plug-flow conditions and TiS values.^9,33^
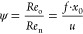
5

### 2.3 Experimental Design

Optimization of the TiS value using input parameters *f* and *x*_0_ was done with a full factorial design of experiments (DOE) using JMP Pro 19 software. Each was given four levels, with all experiments replicated a minimum of three times. Four levels were chosen due to prior knowledge of varying TiS values at a range of oscillatory parameters. A center point was also added, which was tested periodically throughout the study to account for potential variation between different days. Dependent variables derived from the amplitude and frequency in the form of dimensionless numbers calculated through eqs 2–5 (*Re*_o_, ψ, and *St*) enabled statistical regression analysis using JMP Pro 19. A single baffled column was evaluated on TiS number to identify trends with the DOE runs. Additional baffled columns were later added to verify the trends based upon velocity ratio. A single flow rate was used to understand the effect of oscillatory parameters on TiS number. The amplitude and frequency used in this study were chosen based on their combined dimensionless numbers such as the velocity ratio and oscillatory Reynolds number, when operated at a flow rate of 0.0083 L s^–1^. The DOE consisted of a minimum of 66 runs with *f* and *x*_0_ at values = 0.3, 0.8, 1.4, and 2 Hz and 1, 6, 8, and 12 mm, respectively; the central point was 6.5 mm, 1.15 Hz. During experimentation, 12 mm, 1.4 Hz and 12 mm, 2 Hz were not attainable; therefore, *x*_0_ was decreased to 10 mm at 1.4 Hz with 2 Hz being removed. Table 2 below gives the range of each oscillatory parameter, dimensionless numbers, flow rate, and mean residence times of runs within the DOE. Collected data were processed using MATLAB for deconvolution and TiS number calculation before being statistically analyzed within JMP Pro 19.

**Table 2:** Experimental Range of Independent Variables Amplitude and Frequency, the Respective Range of Dimensionless Numbers, Flow Rate, and Range of Mean Residence Times Attained

condition	range
amplitude (mm)	2–12
frequency (Hz)	0.3–2
oscillatory Reynolds number	75–4189
Strouhal number	0.27–3.18
velocity ratio	0.28–15.93
flow rate (L s^–1^)	0.0083
mean residence time (s)	59–185

Input parameters and their interaction were statistically analyzed to gauge if they are significant predictors for the response function, i.e., TiS number. Second, outliers found within the data set were replicated based on the standard deviation and quartile range of the distribution. Finally, two models using the partial least squares methodologies were produced, one looking at independent input parameters and a second with dependent parameters as predictors for output response TiS number.

The mathematical model, which relates factors and response, was a typical regression problem as in eq 6
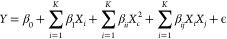
6where *Y* is the response, in this case TiS, *X*_*i*_ is the amplitude, and *X*_*j*_ is the frequency, where *i* and *j* are the index numbers of the interaction pattern *K*. The effects and interactions are denoted as β_*i*_, β_*ii*_, β_*ij*_, and β_*iii*_, where *i* is first order, *ii* denotes quadratic, *iii* represents cubic, and *ij* is the interaction factor. β_0_ and ϵ are the intercept and error terms, respectively. The accuracy of the model is evaluated on the *R*^2^ value, which determines the degree to which the model fits the data. The first model for the independent parameters fitted with a cubic polynomial regression model attained an *R*^2^ of 58% with a root mean square error (RMSE) of 1.69 based on 89 observations. With the analysis of variance, the *F*-test found a probability of *F* < 0.001, indicating a very strong correlation between factors and response. However, as the model fit was quite low, further trends using dependent variables were selected. The *P*-value for each parameter within this model was below the significant threshold of 0.05. The interaction between amplitude and frequency was the most significant factor, with a *P*-value of <0.0001. The *R*^2^ of this model could be improved by replicating experiments several times. However, as amplitude and frequency cannot be directly used as a prediction parameter for TiS when scaling up, it was decided to develop a prediction model using the dimensionless variables. This is because when considering the dimensionless variables, the reactor geometry and net flow rate are included, making it a more suitable scale-up predictor.

**Figure 2:**
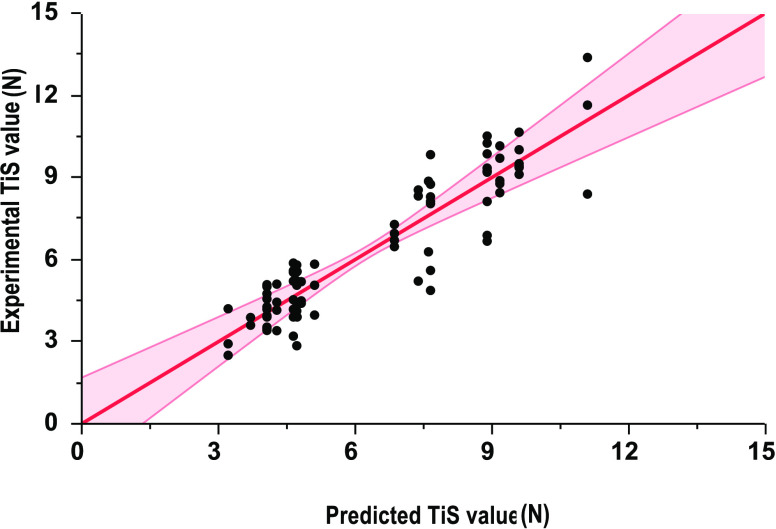
Parity plot of the experimental TiS value against the predicted TiS values *N*. Predicted vs experimental resulted in an *R*^2^ of 81% for a single baffled column. The band represents a 95% confidence fit band of the predicted against the experimental results.

The second model was developed following the same methodology as above using the dependent variables *Re*_o_, ψ, and *St*, similar to the approach as Ahmed et al.^30^ Dimensionless numbers were calculated using eqs 2–5, and a model was developed to predict the TiS value with an *R*^2^ of 81% and a root mean square error (RMSE) of 1.10 based on 89 observations. Again, the *R*^2^ value is suitable for this investigation but could be improved by replicating runs. Outliers have been identified in certain DOE runs and have skewed the model; therefore, replication of these runs could drive the correlation coefficient to higher values, but this is out of scope for the purpose of this study. The *F*-test found the probability of this model to be <0.001, implying a strong correlation between the factors and response. The *P*-values for each factor are presented in Table 3. Any *P*-value above 0.05 was removed from the model unless required for significant hierarchy terms denoted as ∧ in Table 3. Figure 2 shows that a substantial proportion of data lies within the confidence fit shaded red within the figure. This implies the prediction model is satisfactory in predicting TiS using dimensionless numbers. The predictive equation from model 2, which uses the dimensionless to predict TiS, is presented in eq 7.
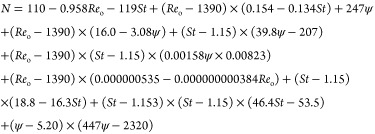
7

**Table 3:** *P*-Values for the Cubic Polynomial Regression Model for Predicting the TiS Value based on Dependent Variables

source	*P*-value	
*St*	0.00000	
*St*^3^	0.00000	
*St*^2^	0.00000	∧
*Re*_o_ × ψ × *St*	0.00000	
*Re*_o_^2^	0.00000	
*Re*_o_ × ψ	0.00001	∧
ψ^2^	0.00002	
*Re*_o_	0.00004	∧
ψ	0.00006	∧
*Re*_o_^3^	0.03976	
*St* × ψ	0.48951	∧
*Re*_o_ × *St*	0.54079	∧

### 2.4 Residence Time Distribution Experimental Setup

For tracer experiments, 0.005 L of potassium hydroxide (KOH) solution (10 mM) was injected 200 mm upstream of the reactor entrance, followed by a 0.04 L water flush. Due to the volume of the reactor, tap water was used as the bulk fluid and fed from the tap into a buffer tank before being pumped into the reactor to negate any impact on the flow from the water pressure. All experiments were conducted at room temperature (21 ± 2 °C). Injection of both tracer and water flush was performed over a 3.5 s period. Due to the distance between the tracer injection point and the reactor inlet, some tracer dispersion may occur. Therefore, a deconvolution to remove the dispersion between these points was conducted through deconvolution in the time domain using the inverse fast-Fourier transformation of the inlet signal from the outlet signal to obtain the true outlet signal. Two pH probes (Mettler Toledo LE407) with a measured response time of 10 s were connected 50 mm from the inlet and outlet of the reactor and controlled through an Arduino. Data were live-streamed into Excel using data streamer software at an interval of 1 s, with the value converted into a concentration of hydroxide ions, as in the study by Abbott et al.^9^ Each experiment was run until the pH returned to its starting value.

### 2.5 Deconvolution Using Fast-Fourier Transformation

Instantaneous pulse injections at *t* = 0 are only possible in ideal scenarios due to the short lag period before the tracer enters the vessel. This, in turn, creates a degree of tracer dispersion uncaused by the reactor itself, affecting the final measured RTD curves; hence, the outlet concentration cannot be directly used to derive the TiS number. Therefore, sensors were placed at both the inlet and the outlet of the vessel to equate the real-time-dependent concentration within the vessel through calculated deconvolution shown in eqs 8–10.
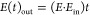
8where *E*(*t*)_out_ is the outlet response, *E*_in_ is the inlet response, and *E* is the real outlet response. The inverse fast-Fourier transformation is used to convolve the product through eq 10 and is used to transform the function to obtain the real RTD in the vessel.
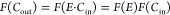
9
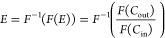
10where *C*_in_ and *C*_out_ are the normalized concentration of hydroxide ions at the inlet and outlet of the OBR and *E* is the real normalized outlet concentration.

### 2.6 Residence Time Distribution (RTD) Analysis

TiS is calculated through normalized RTD curves obtained during tracer experiments and after deconvolution using eqs 11–16 as in previous studies,^9,26,30^ from collected hydroxide ion concentration data. Concentration data are required to generate the RTD curves; therefore, pH is converted into OH hydroxide ion concentration using eq 11.
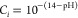
11
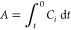
12
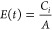
13
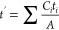
14
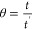
15
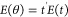
16where pH is the measured value at each probe, *C*_*i*_ and *t*_*i*_ are hydroxide ion concentrations in mM and time at point *i*, *A* is the area under the curve when *C*_*i*_ vs *t*_*i*_ is plotted, *E*(*t*) is the residence time distribution, *t*′ is the mean residence time, θ is the dimensionless time, *E*(θ) is the normalized residence time distribution, and *N* is the number of tanks-in-series. The variance is also used to determine the distribution of the tracer in the vessel and is calculated through the normalized variance as in eq 17. In an ideal plug flow reactor, there is no dispersion of the tracer within the reactor, and thus σ_θ_^2^ = 0.
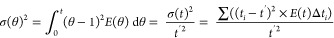
17

Therefore, using the TiS model, the level of plug flow within the OBR can be quantified by calculating the number of CSTRs in series (*N*). If σ(θ)^2^ measures the dispersion from the mean residence time, *E*(θ) is the exit age distribution or normalized mean residence time distribution, and Δ*t*_*i*_ is equal to 1, then the number of TiS can be derived from eq 17 as per eq 18.
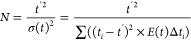
18

The TiS model allows quantification for CSTRs in series.^26^ As *N* increases, it implies the flow tends toward plug flow. Mixed flow will occur at values of *N* < 3. Plug flow is theoretically reached when *N* becomes infinite, but adequate plug flow is achieved at *N* > 10.^9^ Within the proposed OBR, each interbaffled zone can act as a compartment for a single CSTR; this setup consists of 12 interbaffled zones in a single column and 60 in the entire system. η is used to approximate the efficiency of the TiS number to the theoretical maximum as in eq 19.
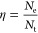
19where *N*_e_ and *N*_t_ are the experimental *N* value and theoretical *N* value based on the number of interbaffled zones within the system and η is the ratio between the two.

Evaluation of the TiS number across the entire reactor was conducted in an equivalent manner to that discussed above and measured along five different locations. These locations are taken after each U-bend along the five-column reactor and identified either as the lower U-bend (LU1) or the higher or upper U-bend (HU2). The number was then ordered from left to right when, as shown Figure 1a.

## 3 Results and Discussion

### 3.1 RTD Curves

An injected pulse of the KOH tracer was fed into the reactor, and the measured pH value was converted to hydroxide ion concentration to produce concentration profiles. Each concentration value was normalized before deconvolution in the time domain through fast-Fourier transformation (FFT) to remove dispersion occurring between the injection point and inlet of the reactor to produce the real outlet function.

**Figure 3:**
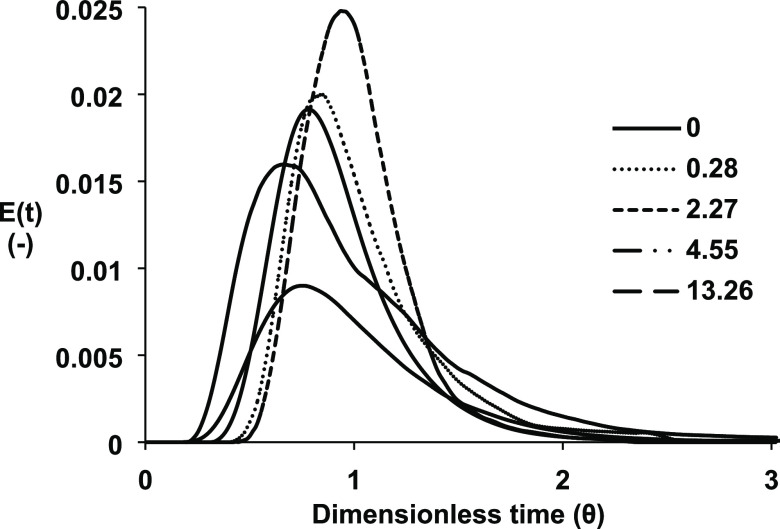
*E*(*t*) time profiles for a single-column oscillatory baffled reactor showing a variation in the residence time distribution when varying the velocity ratio.

Corresponding deconvoluted normalized RTD *E*(*t*) curves are shown in Figure 3, indicating the effect of varying velocity ratios ranging from 0.28 to 15.93. In an ideal scenario, plug flow conditions occur when the outlet signal becomes symmetrical around the mean residence time (θ = 1).^9^Figure 3 shows that a much larger axial dispersion occurs with a velocity ratio of 4.55 than at a velocity ratio of 2.27. When evaluating the number of TiS, the maximum achieved was with a velocity ratio of 2.27 (*x*_0_, 8 mm; *f*, 0.3 Hz) and the lowest at a velocity ratio of 13.26 (*x*_0_, 10 mm; *f*, 1.4 Hz). Additionally, the curves become nonasymmetric around (θ = 1) with peak tailing when the velocity ratio is >4.55; this indicates the degree of back-mixing caused by the reverse stroke of the piston during oscillation, causing a loss of plug flow characteristics at this range.^43^ Several studies have looked at the velocity ratio effect on the TiS number for OBR systems and found that the ideal range for maximizing this value is between 2 and 4,^35^ although a range between 2 and 10 has also been reported to reach near-plug-flow conditions.^7^

Before running the TiS model in full, all results were normalized, followed by deconvolution, by removing the measured inlet results from the measured outlet to provide the real outlet function of the reactor.

**Figure 4:**
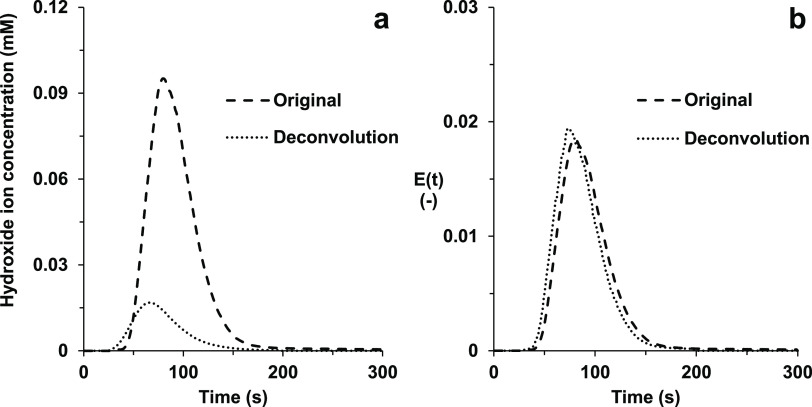
Deconvolution graphs: (a) hydroxide ion concentration; (b) normalized data set.

Deconvolution was conducted in the frequency domain by fast-Fourier transformation (FFT), as shown in eqs 9 and 10. Some issues included the loss of intensity (Figure 4a) and large amounts of noise added to the transformed outlet function (*E*), similar to that reported in the literature.^37−39^ The absence of a curve smoothing function resulted in deconvoluted peaks with major noise on both tails. When a curve smoothing function was applied, the noise could be removed to allow a simple and fast deconvolution processing methodology. Simple curve smoothing through the Savitzky–Golay filter before and after deconvolution and data normalization before deconvolution was suitable for smooth *E* signals with little noise and no loss in intensity. When running a deconvolution with a normalized data set, the resultant *E* curve (Figure 4b) increases the maximum peak height slightly with a minor peak shift due to the removal of mixing prior to the OBR entry.

### 3.2 Effect of Operation Amplitude and Dimensionless Numbers on the TiS Number

**Figure 5:**
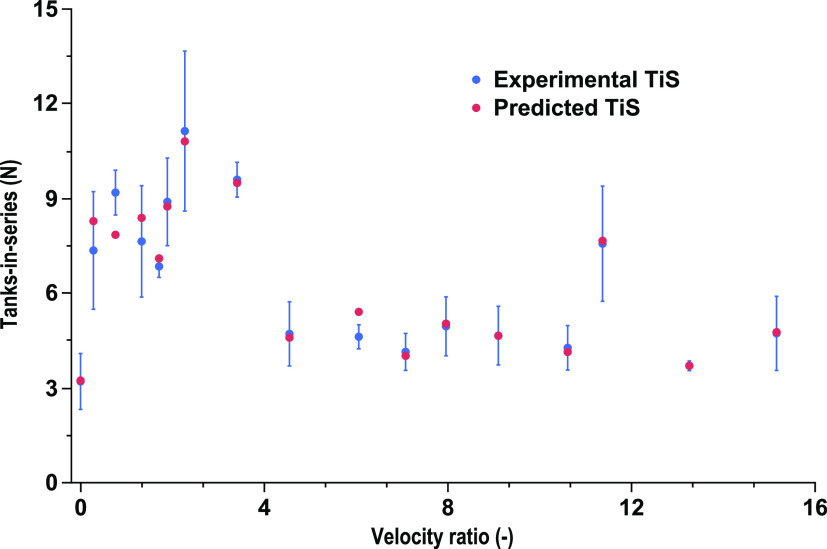
Scatter profile of predicted TiS from eq 7 and the experimental TiS against velocity ratio.

Parameters selected for the DOE to achieve near-plug-flow conditions were based on that reported in the literature.^9,33^ A velocity ratio greater than 1 is needed to ensure full flow reversal; otherwise, no vortices are produced on the downstroke.^7^ However, to maintain a full DOE, velocity ratios below one were used to note any influences as it is often discussed that variables such as *Re*_o_, *St*, and ψ for a continuous OBR system can be used at any scale to predict the range in which the TiS is maximized.^10^ In this particular system, the spacing of baffles moves away from a conventional 1.5 *D* to 1.8 *D* to maximize mass transfer reported by Ni and Gao.^40^ Additionally, a tighter baffle constriction ratio (20%) compared to the standard (25%) was used, giving more reason to include these experimental runs. The velocity ratio against the TiS number is shown in Figure 5. As the velocity ratio drops below 2.27, the TiS value begins to decrease. Similarly, as the velocity ratio begins to increase above 2.27, the TiS number begins to decrease before leveling out at a relatively constant value. This result is similar to that from the study conducted by Ahmed et al., who found that when scaling a single column through length and tube diameter, an increase in the velocity ratio signifies an increase in the oscillation intensity and therefore the vortex size, causing *N* to decrease.^30^ Compared with this study, as the flow rate is fixed, the velocity ratio increase is proportional to *Re*_o_; hence, as either *x*_0_ or *f* increases, the velocity ratio also increases. Therefore, past a velocity ratio of 2.27, the TiS begins to decrease, which coincides with an increase in axial dispersion.^33,42^ The fluid kinetics which explains the decrease in TiS, and therefore an increase in axial dispersion relates to both the axial and radial mixing within the reactor. As stated by Smith et al., a minimum axial dispersion is achieved at a certain *Re*_o_, where the vortices created by the net flow and fluid oscillation redistribute the tracer in a radial direction only. Any further increase in the oscillation intensity will increase the axial dispersion as well as radial dispersion, indicating that oscillation is the predominant variable of axial dispersion.^25^ The TiS number is unusually high at velocity ratios of 0.76 and 11.37, corresponding to *x*_0_ and *f* of 1 mm, 0.8 Hz and 6 mm, 2 Hz, respectively. When the flow regime has no oscillatory motion applied (ψ = 0), a TiS value of 2.92 is achieved, acting as one fully mixed vessel, indicating that the addition of oscillatory motion within the tested range will tend toward plug flow conditions; this correlates with earlier studies where similar results were experienced.^44^ Although the experimental range of TiS values within this part of the report lies close to the minimum for plug flow within OBRs, there is still a clear trend depicted, as shown in Figure 5. The scope of this investigation aims to identify the region of oscillatory parameters that maximize TiS values, including the operational parameters, amplitude and frequency, and their respective velocity ratios for an OBR operated under continuous conditions. This was then used to investigate how U-bends and additional columns affect TiS values in these regions and how these observations compare with the literature. The trend found in Figure 5 is verified within this report in Section 3.3 to determine how a selection of velocity ratios in a single column would translate in a larger system for experimental TiS values. The predicted model TiS values are also shown within the figure to show how they compare against the experimental results.

**Figure 6:**
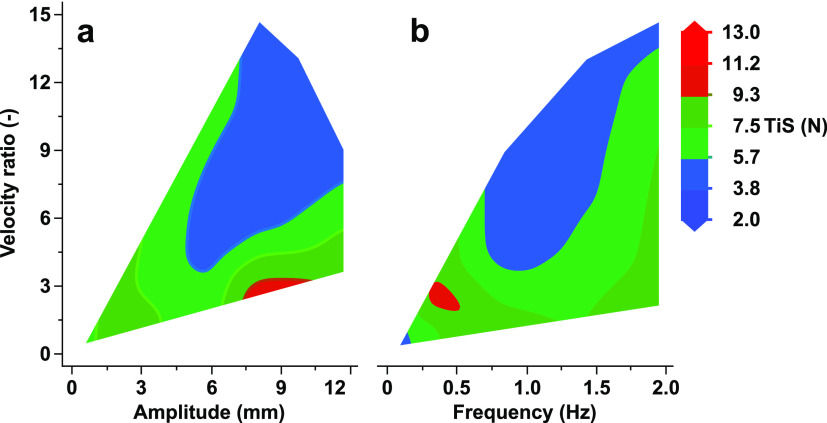
Experimental TiS number (*N*) as a function of velocity ratio: (a) against amplitude; (b) against frequency.

When using a constant bulk fluid at a constant flow rate, the velocity ratio is proportional to *Re*_o_ and, therefore, the product of *f* and *x*_O_, as shown in eqs 2, 4, and 5. In this case, two contour plots are shown in Figure 6, where the TiS number is shown as a function of ψ and either *x*_O_ or *f*. Figure 6a shows the entire range of frequencies for each respective amplitude on the *x*-axis used within the experimental design shown in Table 2, i.e., at an amplitude of 6 mm, all frequency values (0.3, 0.8, 1.4, and 2 Hz) are included. Similarly, Figure 6b contains the entire range of amplitudes for each respective frequency on the *x*-axis used within the experimental design shown in Table 2, i.e., at a frequency of 0.3 Hz, all amplitude values (1, 6, 8, and 12 mm) are included. There are specific regions in Figure 6a, where the TiS number increases mainly as *x*_O_ increases and the ψ remains below 5, indicated by the bright red region. This finding is in contrast to the commercial DN6 and DN15 OBR reported in a study conducted by Oliva et al., where higher *x*_O_ broadens the RTD responses due to the increased back-mixing from oscillation backstrokes.^43^ This can be explained by the larger spacing between baffles (1.8 *D* against 1.5 *D*), allowing longer piston strokes and eddy propagation without the dispersion of the tracer into adjacent baffled sectors. This allows higher amplitudes to attain plug flow characteristics while the net flow controls the movement of the tracer.^42^ Furthermore, high TiS values are achieved when *f* is at the lower end of the experimental range corresponding to ψ near 2. The TiS number is maximized around a value of ψ = 2, and hence high *x*_O_ (8–12 mm) appears to produce higher TiS numbers. There is also a region at both low ψ (<1) and *x*_O_ (<3 mm), implying there could be increased TiS values at lower *x*_O_, provided a ψ = 2 can be achieved through adjustment of *f*. This would need further investigation to confirm. It is noted that although maximizing plug flow conditions may appear optimal, they may not be suitable for all reactor applications, in this case, bioprocesses. For example, on the one hand, in a study where enzymatic hydrolysis is conducted, high velocity ratios compared to this study were used to ensure adequate mixing and full suspension of solids occurred.^45^ On the other hand, high velocity ratios imply higher shear stress rates on cellular cultures, which could be detrimental during cultivation.^16^

As discussed, a lower *f* (<0.5 Hz) implies a higher TiS (>8), as shown in Figure 6b, as indicated by the bright red region in the bottom left figure. The issue with running low *f* within OBR systems concerns the possibility of fouling or dead spots occurring due to the lack of energy to fully suspend particles, for example, in crystallization reactions.^45^ In theory, provided ψ remains around the value of 2, a higher *f* can be used to maximize the TiS value. A similar observation can be made for *x*_O_. Other studies find that the impact of *f* on the tracer dispersion is uncorrelated and has more of an influence on RTD skewness or tailing^43^ but improved mixing.^42^

**Figure 7:**
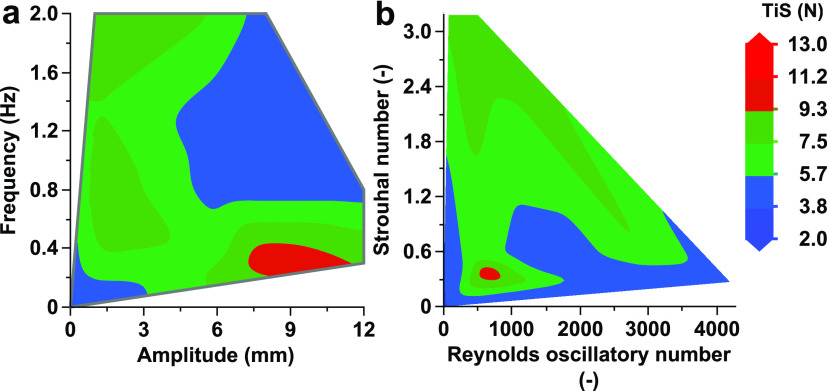
Experimental TiS number (*N*) contour plots as (a) function of amplitude and frequency; (b) function of oscillatory Reynolds number and Strouhal number.

Observing any synergistic impact of *x*_O_ and *f* on the TiS number provides limited results. When comparing their contribution to ψ and their TiS number, some regions tend toward plug flow conditions, such as the lower *f* (<0.5 Hz) and higher *x*_O_ (>6 mm), as shown in Figure 7a. The main findings from this figure are that as ψ nears 2, the TiS value increases, favoring a high *x*_O_ (>6 mm) and low *f* (<0.5 Hz). However, there are other regions of combined parameters that have some degree of plug flow conditions. There is a payoff between the mixing intensity required and the level of plug flow for the application.

In Figure 7b, a lower value of *St* provides a high TiS number. When calculating high Strouhal values with eq 3, it requires a small *x*_O_. To then reach ψ near 2 requires *f* > 2 at the current flow rate. Figure 7b does show that with a *Re*_o_ between 500 and 1500, corresponding to ψ between 2 and 6, tends toward plug flow conditions for this reactor system.

### 3.3 Change of RTD across the Reactor Length

**Figure 8:**
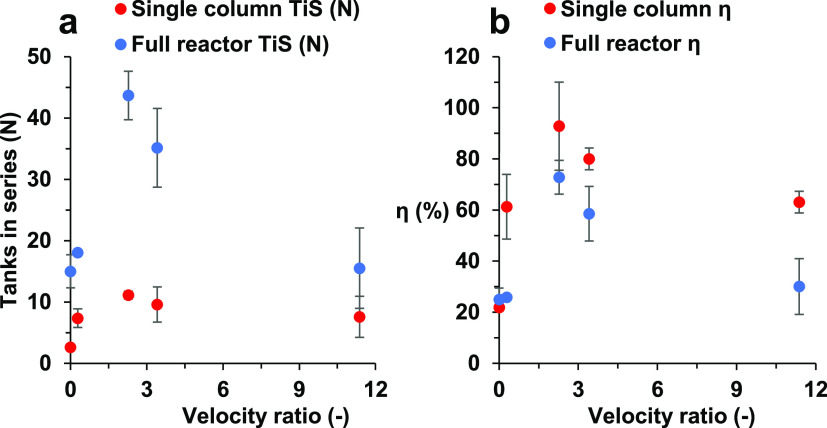
Flow characterization plots using the TiS model in two different setups, single column (red points) and five consecutive columns (blue points) with the velocity ratio against (a) experimental TiS numbers and (b) the percentage of experimental TiS number η (%) achieved against the theoretical maximum.

**Figure 9:**
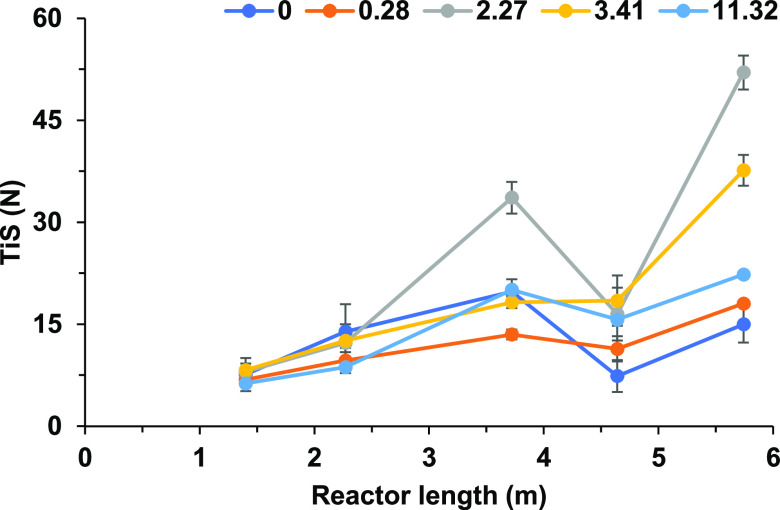
TiS value measured at separate locations along the OBR for the full five-column reactor.

It is stated that plug flow is achieved when *N* > 10.^22^ The theoretical maximum TiS is equal to the number of interbaffled regions within the column; in this case, it is 12. In this report, a single column reached a maximum TiS of 13.38 at 8 mm and 0.3 Hz. The explanation of the TiS exceeding the theoretical maximum is likely due to the small distances between the inlet and outlet probes of the reactor acting as mixing vessels, similar to that described in the connection of two baffled columns in another study.^9^ Also, with the majority of TiS numbers below the threshold of plug flow conditions, it could be perceived that the tested experimental setup is insufficient to determine parameters that affect plug flow conditions. An additional four columns were added to the system, as shown in Figure 1a, to validate the trends found in Figure 5. When adding additional columns, five selected velocity ratios were evaluated within the full system, and the RTD was measured initially at the reactor outlet only. TiS results for the full system at the outlet only are found in Figure 8, whereas the TiS at various stages along the length of the reactor is shown in Figure 9 to understand the variation of the TiS number with additional columns, U-bends, and different ψ values.

Figure 8 shows the achieved TiS values (Figure 8a) alongside the percentage against the theoretical maximum (Figure 8b) with two different setups: a single column and the full five-column connected system. Percentage η (%) is used to compare the differences between the two systems while maintaining constant parameters elsewhere. As shown in Figure 8a, the trend for the TiS number relative to the velocity ratio is similar for all ψ. The maximum TiS value closest to the theoretical maximum is at ψ = 2.27, achieving an average total system *N* = 43.67. Compared with ψ = 0, in which no oscillatory motion is applied, both single column and full system TiS numbers are lower, reaching an average of *N* = 14.99 in the full system, confirming the previous observation that the application of oscillatory motion will tend to plug flow conditions. This is due to the lack of vortex formation in the laminar flow regime without oscillatory motion.^46^ Similarly, at a velocity ratio of ψ = 0.28, there is only a slight oscillatory motion placed on the net flow; hence, little to no flow reversal is exhibited and acts like flow with no oscillatory motion, explaining a similar TiS value to that of ψ = 0. In each experiment, the five-column setup achieved a lower percentage η (%) for the single column. Figure 8b shows a loss between the single column and multicolumn system averaging around 20–30%, dependent on the velocity ratio. This is similar to what was experienced in a study conducted by Egedy et al.; however, their findings were a lot more dramatic, with up to 71.4% oscillation dampening experienced after the first U-bend.^47^ It appears that larger losses are experienced when ψ is at the extremes of the tested range. The mixing conditions become more turbulent due to more energy being put into the system from an increase in *Re*_o_.^33^ As discussed earlier, the combination between net flow and oscillatory motion past a certain velocity ratio will increase the axial and radial mixing. Therefore, oscillations are the predominant variable of axial dispersion, forcing the tracer to spread into adjacent interbaffled zones.^25^ This explains why at higher values of ψ, as shown in Figure 8b, a larger decrease in η is shown at ψ = 11.32 compared to ψ = 2.27. The same can be said for significantly low values of ψ, where *Re*_o_ cannot dominate the net flow, and the vortex cycle cannot be realized, causing mixing to be controlled through molecular diffusion alone, and is shown in the data at ψ = 0.28.^35^ A decrease in the TiS value when scaling up is more likely caused by the first U-bend, which immediately creates nonasymmetric eddy formation along the entire reactor system. This sudden change in momentum forces eddies to collide with each other around the U-bend, which, in turn, propagates down both connecting columns. The addition of multiple U-bends and additional baffled sections may also further decrease the TiS value along the reactor. Comparing these results with Figure 5, the velocity ratios used and their respective TiS values in both the single-column and five-column systems compare well. If using the trend from Figure 5 alone, it is expected that the respective TiS numbers for each ψ should ascend from 0, 0.28, 11.32, and 3.41 and maximize at ψ = 2.27. When comparing the experimental results in the full system, the trend is similar to that at ψ = 0 having the lowest average TiS value of 14.99, ψ = 2.27 having the highest value of 43.67, and the second highest value of 35.2 from ψ = 3.41. This set of results aligns exactly with the trend set in Figure 5. As for ψ = 0.28 and 11.37, their TiS values in the full system were 18.05 and 15.52, respectively. According to Figure 5, the finding is in contrast for ψ = 0.28, having the second lowest TiS value; however, when observing the error bar in both Figures 5 and 8, there is an overlap between both values and outliers could be skewing the values.

With five additional columns, the TiS number increases at the outlet due to the increasing number of interbaffled zones within the system, as shown in Figures 8a and 9. The TiS number across the five-column reactor was found to change effects depending on the measured location, as shown Figure 9. The highest TiS value experimentally determined across each section of the reactor was at ψ = 2.27 (8 mm, 0.3 Hz), identifying this to be the optimal set of parameters explored, followed by ψ = 3.41. It is expected for all ψ that the TiS value should linearly increase as the length of the reactor increases, similar to that which occurs within Figure 9 for a velocity ratio of 2.27. It shows a steady increase in the TiS value as the interbaffled zones increase, either by adding additional columns or extending the column length. However, the measurement point in higher U-bend 1 (HU1) gave only a very slight increase in the TiS value against its previous measurement location (LU1), which goes against the expected linear trend. HU2 exhibits an unusual phenomenon where even though the tracer will have passed through more interbaffled zones (36 interbaffled zones at LU2 and 48 interbaffled zones at HU2), the TiS value is reduced for most ψ. If the tracer has maintained plug-flow-like conditions, the TiS value should have increased linearly at each measured location. However, the data suggest that between LU1 and HU1 and LU2 and HU2, there is a significant increase in axial dispersion as the TiS value has not increased. Furthermore, there must have been a significant increase in axial dispersion between LU2 and HU2 due to the decrease in the TiS value between each location for the tested values of ψ.

The varied axial dispersion that occurred along the reactor length could be due to several reasons. The lack of increase in the TiS number between LU1 and HU1 is most likely attributed to the U-bend geometry in LU1 and the entry into HU1. In both LUs, there is a lack of baffles within along with several locations for tracers to stagnate. It is well documented that baffles promote mixing within the tube, forcing vortex rings to form downstream of the baffle.^24,33,48^ On each backstroke, the flow is then reversed, sweeping the vortex into the middle of the channel before the cycle repeats on the next oscillatory phase.^49^ In a tube that lacks baffles, the vortex formation will therefore not occur and radial mixing becomes dependent on molecular diffusion and axial mixing on the net flow, leaving areas for the tracer to build up. Additionally, the tracer moves slower around the outside radii of the U-bend with a lack of baffles and faster on the shorted internal radii of the U-bend as visually observed by Mackley and Ni using flow visualization experiments.^46^ A similar scenario occurs at the entry to HU1, in which there is a large gap between the final column baffle and the 90° bend. Commercial OBRs such as the DN15 and DN6 used in other reports contain baffled U-bends, which are likely to maintain the near-plug-flow characteristics along the reactor length.^12,43,50^ Another explanation for a dramatic drop in TiS at HU2 is due to the measurement ports and membrane ports. As the fluid enters from the riser column into HU, it hits an area where reduced mixing may occur, as shown in Figure 1a. In a single-phase operation, the gap is filled with liquid, whereas when operated for its purpose, the gap is filled with excess gas to be removed. It is highly likely due to the lack of crossflow or vortex formation around these locations that the tracer stagnates and only removes itself due to molecular diffusion. Once out of the HU, the tracer enters back into the cycle of vortex formation on either side of the baffles and maintains a near-plug-flow behavior.

**Figure 10:**
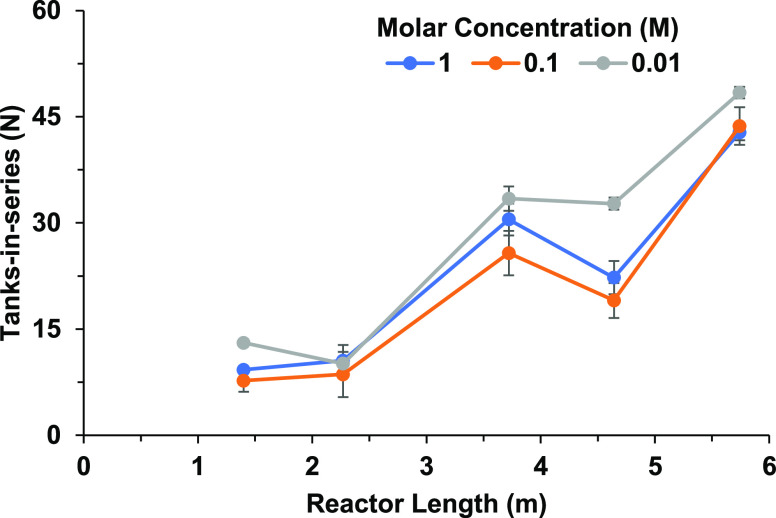
TiS value along the reactor length using different molar concentrations of the tracer.

Another explanation refers to the method in which TiS data were collected. The fact there are risers and downers in this setup could mean gravity provides an additional back-mixing mechanism to the tracer. When selecting tracers, it is very important to ensure the physical and chemical properties have no bias involvement with the bulk fluid.^51^ A small set of duplicated experiments were run using different molar concentrations of the tracer (1, 0.1, and 0.01 M) using ψ = 2.27, with other parameters remaining the same. Figure 10 shows that the density of the tracer has no significant impact on the trend of the TiS value along the reactor length.

**Figure 11:**
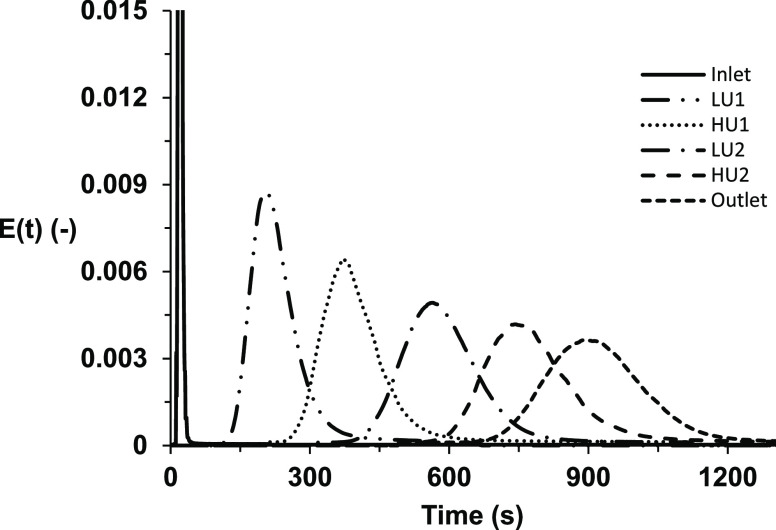
RTD curves at separate locations for the parameters that achieve a maximal TiS value within the data set (five-columns with ψ = 2.27, *x*_O_ = 8 mm, and *f* = 0.3 Hz).

Figure 10 shows a similar pattern to that shown in Figure 9, where there is a slight difference in the TiS value from the measurement point LU1 to HU1 and a decrease in the TIS value from points LU2 and HU2. This indicates that the density of the tracer has no significant impact on the TiS values within the tested range. Apart from the concentration profiles increasing as the molar concentration increases, there is a minor variation in the TiS number at each point along the reactor length. Surprisingly, the lowest molar concentration of 0.01 M produced the highest TiS value of 48.39 at the outlet, implying that even at extremely low concentrations, a RTD profile can be determined. Ni et al. also investigated the impact of tracer density on the RTD profile within an OBR with geometry similar to this study. They reported that, overall, the density of the tracer was independent of axial dispersion within the tested range. However, they also found, but could not explain, a slight increase in axial dispersion when increasing the tracer at the lower ends of the tracer density range tested, whereas the results reported in this study found that a lower tracer concentration resulted in an increase in TiS values. If taking the range of error bars into the discussion, it could be argued that the lower the concentration of the tracer, the higher the TiS value produced; however, the trend of TiS at different points along the reactor remains the same. The RTD curves for each location are depicted in Figure 11. The Gaussian distribution of the RTD curves exhibited limited axial dispersion away from the center line of each *E*(*t*) curve, implying near-plug-flow conditions along the reactor length. These plots are similar to figures made of other multipass OBR setups, exhibiting minimal axial dispersion.^12,33^

### 3.4 Scaling the OBR for Bioprocesses

Many chemical reactions can take several hours to complete, which are feasible in small OBR systems such as the commercial COBR15.^12,43,50^ However, bioprocesses can take several days, which would require impractical lengths in conventional tubular reactors for continuous processing where mixing is reliant on turbulent fluid flow.^16^ OBR systems are known to disassociate the mixing from the net flow and enable uniform mixing throughout, providing ψ is above 1.^18^ Therefore, achieving these long residence times is more realistic with an OBR compared to conventional continuous flow technologies. An OBR design methodology proposed by Stonestreet and Van Der Veeken discussed how reaction kinetics can be understood within a batch OBR first to identify the reaction residence time. Followed by RTD experiments to identify optimal dimensionless numbers to maximize plug flow characteristics, both these data sets can be combined and translated into a larger commercial scale system. Using the dimensionless number velocity ratio, the reactor length can then be determined by selecting a column diameter and combining reaction residence time with mean residence times found in the RTD experiments.^35^ In the OBR used in this study, one could design a reactor of known length and adjust the flow rate so that the mean residence time is equal to the reaction residence time while maintaining a velocity ratio of 2.27 using the oscillatory Reynolds number. The OBR studied in this report reached a minimum mean residence time of 900 s at ψ = 2.27 and *Re*_n_ = 263 in the full five columns. This was calculated from eq 14, which is 7% higher than the hydraulic residence time. This is similar to a study by Reis et al. with a difference of 32.5% between the mean and hydraulic residence time, whereas Phan and Harvey identified the mean residence time to be 8.5% higher than the hydraulic residence (hydraulic residence time is crudely calculated from the reactor volume divided by flow rate).^22,52^ For a bioprocess with a 2-day residence time, this would require a reactor of 1420.8 m, assuming mean residence time scales linearly, and the same flow rate is used. The reactor length of this magnitude is likely infeasible due to oscillation dampening. Therefore, bioprocesses using OBRs should either operate as single batch columns, or system recirculation units, or reduce the flow rate with a moderate reactor length increase.

Although this study did not investigate the impact of net flow on scale-up feasibility, several studies have investigated the combination of dimensionless numbers, including *Re*_n_, with axial dispersion. Abbott et al. found that with an increasing flow rate of *Re*_n_ = 73–259, there was an increase in the TiS value recorded.^9^ Similarly, Stonestreet and Van Der Veeken observed the same trend when increasing the *Re*_n_ between 95 and 252.^35^ They explained that at *Re*_n_ = 100, the vortex size was significantly larger than that at *Re*_n_ = 10. It appears that the TiS number increases with the net flow as stated in both reports within their tested ranges of net flow. However, increasing TiS by increasing *Re*_n_ is limited to a point, as once the net flow reaches the turbulent region, both baffles and oscillatory motion will have little effect on axial dispersion.^46^ A more recent study by Briggs et al. found that *Re*_n_ had little impact on the axial dispersion coefficient,^12^ which contradicts the studies aforementioned by Abbott et al. and Stonestreet et al. It may appear that rather than influencing axial dispersion, the net flow should control the mean residence time only, and the oscillatory Reynolds number should control the mixing and maintain the desired velocity ratio.

Studies have concluded that a minimum net Reynolds number of 50 is required to achieve convection.^35^ However, high levels of plug flow have been achieved at values as low as *Re*_n_ = 10 in smooth periodic and helical baffled systems,^36,52^ albeit the scale is significantly smaller. Flow rates resulting in *Re*_n_ < 10 report that *Re*_o_ has little impact on axial dispersion,^22^ with axial dispersion through baffled sectors increasing due to lack of transport along the reactor length.^42^ Furthermore, a minimum net oscillatory Reynolds number of 130 must be met to achieve near-plug-flow conditions alongside adequate mixing within a continuous OBR,^53^ which could incur very high velocity ratios at these ultralow flow rates. In a study conducted by Slavanić et al. using low *Re*_n_, they found that when high *x*_0_ and low *f* are used to reach a higher *Re*_o_, the fluid operates with low levels of axial dispersion and with adequate mixing,^53^ providing ψ > 1.^54^ The current research found a trend of higher TiS values at *x*_0_ (8–12 mm) in the single-column experiments compared to values below 6 mm. Thus, the five-column system design could prove beneficial for use as a bioreactor as lower flow rates can be used while reaching adequate mixing and longer residence times.

It is important to understand the reaction kinetics within an OBR before designing a reactor due to the potential impacts of their enhanced mixing properties. Additionally, OBR mixing benefits from low power requirements when maintaining a velocity ratio between 2 and 4 compared to CSTRs.^16,55^ Bioprocessing products vary in value dramatically, with many manufacturing processes requiring large volumes between 2 × 10^4^ and 2 × 10^5^ L in STRs with scaling factors between thousands to millions from lab to commercial scale to make them economically practical.^56^ The only reported commercial pilot OBR for bioprocessing is up to 25 L, and although the production throughput was dramatically increased,^57^ either one large-scale system or multiple small systems would be required to reach commercial outputs. A second issue with continuous bioprocesses in OBRs is the lack of aeration and feed points and oscillation dampening caused by either aeration, suspended solids like cells, momentum changes, and friction through the numerous baffled constrictions.^16^ Scaling OBRs to pilot plant and production plant scales are said to be linear with geometric parameters, providing dynamic numbers (dimensionless numbers) are equal.^10^ Stonestreet and Harvey presented a scale-up methodology for reaching production plant scale using OBR technology but did not comment on the possible limitations of oscillation dampening.^19^ In this case, there are two tangible scale-up strategies when investigating OBRs for continuous bioprocessing, either by increasing the tube diameter as pointed out by Jian et al. and Abbott et al^16,32^ or by extending the reactor length with additional baffled columns as proposed by Stonestreet and Harvey.^19^ However, these scale-up methods may be constrained by fluid oscillations. Observations on the impact of the reactor length on oscillations must be made to avoid oscillations becoming dampened and void at extended reactor lengths. Care must also be taken to ensure the system is operating above the minimum net flow for *Re*_n_ when scaling with the tube diameter.

The recommended lab-scale tube diameter is reported between 15 and 150 mm,^7^ with 150 mm being the recommended maximum for lab-based continuous studies,^25^ although one study of polymerization adopted 380 mm.^10^*Re*_n_ will naturally decrease as the tube widens, as shown eq 4. Bioprocesses will likely require exceptionally long residence times; therefore, scaling through the tube diameter for the volumetric increase would be highly beneficial as lower flow rates can be attained easily. Additionally, bioprocess culture media can start at high viscosities due to glucose concentrations,^58^ or high solid loading contents when using starch-based feedstocks such as bread.^5^ This can lead to the initial *Re*_n_ being lower until the cellular matter has consumed the carbon source and begun to replicate, in turn, slightly increasing the fluid density. Identification of fluid rheology is therefore crucial to maintain efficient mixing at the desired flow rate and tube diameter using ψ as a guide in bioprocesses. For example, if *Re*_n_ = 50 is required, a plot of the minimum flow rate against the tube diameter can be made for a bioprocess, for example, microalgae cultivation. Microalgae broth has a liquid density of 1025 kg m^3^ and a viscosity similar to water ranging from 0.8 to 2.6 mPa s.^59^

**Figure 12:**
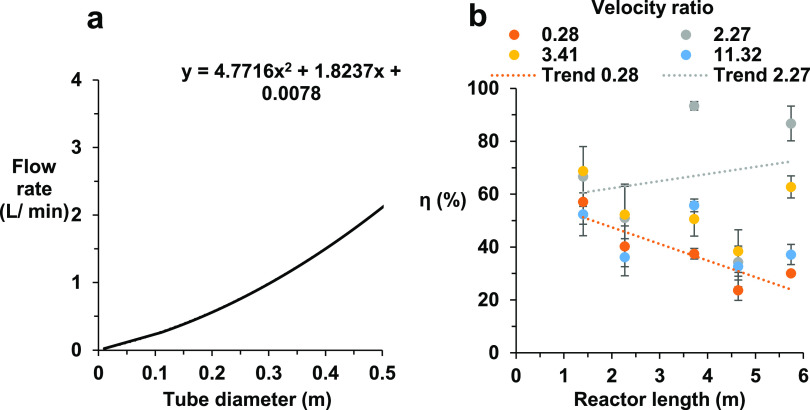
(a) Minimum flow rate required for an OBR to maintain a Reynolds number above 50 at different tube diameters for a microalgae bioprocess. (b) Percentage of the maximum TiS number in comparison to the theoretical maximum TiS at separate locations across the reactor.

Figure 12a shows the plots for the minimum flow rate using the density and viscosity of a microalgae culture at various tube diameters while maintaining an *Re*_n_ of 50 for scaling an OBR. The curve equation shown can then be used to determine the minimum flow at any tube diameter.

Alternatively, scale-up can be produced by adding lengths of baffled columns connected by U-bends. However, this has a limitation on the mixing ability within the reactor at long lengths. Reduction in oscillatory motion because of pressure loss is a critical issue with OBRs as this is the key phenomenon associated with mixing. A numerical study conducted by Mazubert et al. found that the pressure drop for a single orifice OBR equates to 0.79 kPa m^–1^.^60^ Briggs and associates found that extending the length of an OBR will progressively diminish the oscillatory motion as more U-bends and lengths of baffled columns are added. They found that the attained oscillatory amplitude is halved at the outlet when 11 U-bends and 22 columns are used.^12^ From previous computational fluid dynamic (CFD) evaluations (data not shown and unpublished), the U-bend is suspected to have more of an influence on oscillatory dampening than that of the baffle constrictions. This is due to momentum changes and collision of eddies along a nonsymmetrical pathway around U-bend radii and fluid traveling at different distances. Without eddy formation on either side of the baffle, the mixing efficiency will begin to decrease and, in turn, axial dispersion will increase, losing all plug flow characteristics.^44^ Egedy et al. reported similar results, with up to 71.4% oscillation dampening being experienced after the first U-bend connection.^47^ Mackley and Ni, in flow visualization studies, found that at low flow rates, the dispersion was not impacted by the U-bends. This finding leads to the requirement of low flow rates being used when scaling the OBR by increasing the reactor length with U-bends and additional columns.^46^

Figure 12b illustrates the effect of extending the length of the OBR from 1.2 to 5.74 m by adding additional columns after one U-bend. It finds no negative impact on plug flow characteristics that occurred at ψ = 2.27 as the value of η remained consistent between 64 and 68% at each lower measuring point (LU1, LU2, and outlet). Comparable results were found for ψ = 3.41, although the range η was larger (57–42%). These results are consistent with other studies, which have evaluated axial dispersion with OBRs consisting of multiple columns and U-bends. Ni and Pereira’s study of a 14-column OBR, each at 1000 mm in length and geometry similar to the one in this study, reported low axial dispersion coefficients at different locations along the reactor length when operating at higher amplitudes (>7 mm).^33^ As discussed earlier in Figure 9, both HU1 and HU2 showed a significant drop in plug flow characteristics, with η decreasing to between 35 and 40%. Interestingly, at ψ = 0.28 (1 mm, 0.3 Hz), a decrease in the TiS number was experienced, which correlates with the results reported by Briggs et al., where small *x*_0_ values will lose oscillatory motion faster due to having less initial energy input into the fluid to overcome all constrictions and momentum changes.^12^ These results confirm that near-plug-flow conditions can be achieved and maintained at longer reactor lengths when operating at a higher *x*_O_ when scaling OBRs with additional baffled columns.^61^

This work confirms that scaling with both tube diameter and reactor length is possible within OBRs when operating with high *x*_O_ and low *f*. With both scaling strategies discussed, care must still be taken with the OBR design and operational parameters to ensure the desired application and mixing requirements are successfully carried out, as discussed by Avila et al.^7^ The idea of having an OBR for processes with residence times greater than 1 day and minimizing the limitations on mixing efficiencies can be done in certain strategies:

1. Operating long residence times as a batch process in a single column with a desired volume, scaling up by increasing the tube diameter and maintaining desired mixing conditions.^31^
2. Operating a single long column without connecting U-bends with the required net flow and diameter for volume requirements while maintaining the desired mixing conditions based on the dimensionless numbers. However, this route may require infeasible lengths of a column.
3. Operating several smaller systems on a recirculatory batch operation started at different time intervals to have continuous output. The number of smaller systems needed is dependent on the required throughput of the process.
4. Adopting a multicolumn approach with columns in parallel, the desired volume can be controlled by extending the length or adjusting the tube diameter, and mixing is maintained with dimensionless numbers. This will mitigate any effect of the U-bends but may impact eddy propagation from the location of the oscillatory mechanism. It is reported that this type of setup has consistent dispersion patterns in each column.^46^

Using a multipass system as described Stonestreet and Harvey with the desired tube diameter and flow rate based on the reaction kinetics and rheology.^19^ Operating at the desired mixing conditions for that geometric design based on the dimensionless numbers relate to *Re*_n_. The design of the U-bend should be optimized to monitor the impact of ports and valves to minimize disruption to the fluid flow. Low *Re*_n_ should be used to minimize U-bend impact on dispersion as per Mackley et al.^46^

## 4 Conclusions

This study investigated the effect of oscillatory parameters on an OBR system adopted for bioprocess applications. A central orifice baffled column with a diameter of 40 mm, spacing of 1.8 *D*, and constriction ratio of 20% was used. Opportunities and limitations of scale-up were explored regarding oscillatory parameters, U-bends, and reactor sections. The investigation reported that plug-flow-like conditions were achieved over a range of oscillatory parameters between velocity ratios of 1.7 and 3.5. A maximum TiS value of 43.68 was reached in the five-column system compared with a maximum value of 13.38 in the single column. The maximum TiS value was reached at ψ = 2.27 with a trend of increasing TiS near ψ = 2. An example of scaling through the tube diameter was proposed based on previous studies, residence times, and rheological properties of a microalgae bioprocess. The net flow must be kept above *Re*_n_ > 50 to ensure fluid convection, although other studies have achieved plug flow at lower values. The OBR reported here attained a high TiS number with *x*_0_ > 8 mm and *f* < 0.8 Hz. This can allow plug flow to operate at *Re*_n_ < 50 with adequate mixing providing *Re*_n_ < *Re*_o_. This result benefits bioprocesses with long residence times by allowing reduced flow rates while ensuring near-plug-flow conditions.

Connecting U-bends used to scale up the reactor with additional baffled sections had a significant impact on TiS. The U-bend geometry including the lack of baffles and areas for fluid to stagnate, such as probe ports or air vents, dramatically reduced the TiS number along the reactor length. No impact of tracer density was found on TiS along the reactor length. Lower U-bends achieved a higher TiS number than the upper U-bends. The study reported that TiS values maintain a linear increase along the reactor length at oscillatory parameters *x*_0_ = 8 and 12 mm with minimal effect from additional sections and U-bends. This implies the maintenance of plug flow characteristics (η) under these conditions. Alternatively, lower starting *x*_0_ (<6 mm) found a linear decrease in TiS along the reactor length when additional U-bends were introduced, implying a decrease in plug flow conditions (η) at lower amplitudes. In all studied cases of ψ, the U-bend had the greatest impact on reducing the TiS at the first U-bend. The TiS in all cases dropped by 20–30% after the first U-bend compared to the straight column. This phenomenon is thought to be the result of momentum change in the U-bend and collision of eddies propagating back up the column.

The study determines the feasibility of scale-up routes that can maintain near-plug-flow conditions and potentially operate at low flow rates with adequate mixing. Amplitudes >8 mm retained minimal levels of dispersion when additional baffled sections were added. The geometric design of the U-bend played a key role in plug flow characteristics within the reactor. It is concluded that the reported OBR design has the potential for long residence times at scale with continuous aeration, such as in bioprocesses. Further work will be conducted on the U-bend geometry to minimize flow disruption and identify reactor length limits.

## Abbreviations

OBR: oscillatory baffle flow reactor
CSTRs: continuous stirred tank reactors
TiS: tanks-in-series
RTD: residence time distribution
*Re*_o_: oscillatory Reynolds number
*Re*_n_: Reynolds number
*St*: Strouhal number
ψ: velocity ratio
DOE: design of experiments
LU: lower U-bend
HU: higher U-bend

## References

[ref1] Enamala M. K.; Enamala S.; Chavali M.; Donepudi J.; Yadavalli R.; Kolapalli B.; Aradhyula T. V.; Velpuri J.; Kuppam C. Production of Biofuels from Microalgae - A Review on Cultivation, Harvesting, Lipid Extraction, and Numerous Applications of Microalgae. Renewable Sustainable Energy Rev. 2018, 94, 49–68. 10.1016/j.rser.2018.05.012.

[ref2] Ikwebe J.; Harvey A. P. In Intensification of Bioethanol Production by Simultaneous Saccharification and Fermentation (SSF) in an Oscillatory Baffled Reactor (OBR), Institution of Chemical Engineers Symposium Series, Vol. 157, pp 60–65.

[ref3] López-Gómez J. P.; Alexandri M.; Schneider R.; Venus J. A Review on the Current Developments in Continuous Lactic Acid Fermentations and Case Studies Utilising Inexpensive Raw Materials. Process Biochem. 2019, 79, 1–10. 10.1016/j.procbio.2018.12.012.

[ref4] Gnanasekaran R.; Dhandapani B.; Iyyappan J. Improved Itaconic Acid Production by Aspergillus Niveus Using Blended Algal Biomass Hydrolysate and Glycerol as Substrates. Bioresour. Technol. 2019, 283, 297–302. 10.1016/j.biortech.2019.03.107. 30921582

[ref5] Cox R.; Narisetty V.; Nagarajan S.; Agrawal D.; Ranade V. V.; Salonitis K.; Venus J.; Kumar V. High-Level Fermentative Production of Lactic Acid from Bread Waste under Non-Sterile Conditions with a Circular Biorefining Approach and Zero Waste Discharge. Fuel 2022, 313, 122976 10.1016/J.FUEL.2021.122976.

[ref6] Abdel-Raouf N.; Al-Homaidan A. A.; Ibraheem I. B. M. Microalgae and Wastewater Treatment. Saudi J. Biol. Sci. 2012, 19, 257–275. 10.1016/j.sjbs.2012.04.005. 24936135 PMC4052567

[ref7] Avila M.; Kawas B.; Fletcher D. F.; Poux M.; Xuereb C.; Aubin J. Design, Performance Characterization and Applications of Continuous Oscillatory Baffled Reactors. Chem. Eng. Process. 2021, 108718 10.1016/j.cep.2021.108718.

[ref8] Zhang C.; Maruggi G.; Shan H.; Li J.; Evensen O.; Jemielity J.; Li J.; Zhang C.; Maruggi G.; Shan H. Advances in MRNA Vaccines for Infectious Diseases. Front. Immunol. 2019, 10, 594 10.3389/fimmu.2019.00594. 30972078 PMC6446947

[ref9] Abbott M. S. R.; Harvey A. P.; Morrison M. I. Rapid Determination of the Residence Time Distribution (RTD) Function in an Oscillatory Baffled Reactor (OBR) Using a Design of Experiments (DoE) Approach. Int. J. Chem. React. Eng. 2014, 12, 575–586. 10.1515/ijcre-2014-0040.

[ref10] Ni X.; Mackley M. R.; Harvey A. P.; Stonestreet P.; Baird M. H. I.; Rama Rao N. V. Mixing through Oscillations and Pulsations -A Guide to Achieving Process Enhancements in the Chemical and Process Industries. Chem. Eng. Res. Des. 2003, 81, 373–383. 10.1205/02638760360596928.

[ref11] Clarke K. G. Bioprocess Scale Up. Bioprocess Eng. 2013, 171–188. 10.1533/9781782421689.171.

[ref12] Briggs N. E. B.; McGinty J.; McCabe C.; Raval V.; Sefcik J.; Florence A. J. Heat Transfer and Residence Time Distribution in Plug Flow Continuous Oscillatory Baffled Crystallizers. ACS Omega 2021, 6, 18352–18363. 10.1021/acsomega.1c02215. 34308066 PMC8296600

[ref13] Masngut N.; Harvey A. P.; Ikwebe J. Potential Uses of Oscillatory Baffled Reactors for Biofuel Production. Biofuels 2010, 1, 605–619. 10.4155/bfs.10.38.

[ref14] Alissandratos I. Novel Bioprocessing Technologies for the Cultivation of Microalgae. Ph.D. Thesis, Cranfield University: Cranfield, 2019.

[ref15] Plumb K. Continuous Processing in the Pharmaceutical Industry: Changing the Mind Set. Chem. Eng. Res. Des. 2005, 83, 730–738. 10.1205/cherd.04359.

[ref16] Abbott M. S. R.; Harvey A. P.; Valente Perez G.; Theodorou M. K. Biological Processing in Oscillatory Baffled Reactors: Operation, Advantages and Potential. Interface Focus 2013, 3, 20120036 10.1098/rsfs.2012.0036. 24427509 PMC3638279

[ref17] Bianchi P.; Williams J. D.; Kappe C. O. Oscillatory Flow Reactors for Synthetic Chemistry Applications. J. Flow Chem. 2020, 10, 475–490. 10.1007/s41981-020-00105-6.

[ref18] McDonough J. R.; Phan A. N.; Harvey A. P. Rapid Process Development Using Oscillatory Baffled Mesoreactors - A State-of-the-Art Review. Chem. Eng. J. 2015, 265, 110–121. 10.1016/j.cej.2014.10.113.

[ref19] Stonestreet P.; Harvey A. P. A Mixing-Based Design Methodology for Continuous Oscillatory Flow Reactors. Chem. Eng. Res. Des. 2002, 80, 31–44. 10.1205/026387602753393204.

[ref20] Mortazavi H.; Pakzad L. The Hydrodynamics and Mixing Performance in a Moving Baffle Oscillatory Baffled Reactor through Computational Fluid Dynamics (CFD). Processes 2020, 8, 1236 10.3390/pr8101236.

[ref21] Zheng M.; Mackley M. The Axial Dispersion Performance of an Oscillatory Flow Meso-Reactor with Relevance to Continuous Flow Operation. Chem. Eng. Sci. 2008, 63, 1788–1799. 10.1016/j.ces.2007.12.020.

[ref22] Phan A. N.; Harvey A. Development and Evaluation of Novel Designs of Continuous Mesoscale Oscillatory Baffled Reactors. Chem. Eng. J. 2010, 159, 212–219. 10.1016/j.cej.2010.02.059.

[ref23] Sutherland K.; Pakzad L.; Fatehi P. Comparison of Mixing Performance between Stationary-Baffle and Moving-Baffle Batch Oscillatory Baffled Columns via Numerical Modeling. Chem. Eng. Commun. 2022, 17–46. 10.1080/00986445.2020.1823841.

[ref24] Ni X.; De Gélicourt Y. S.; Baird M. H. I. I.; Rao N. V. R. R. Scale-up of Single Phase Axial Dispersion Coefficients in Batch and Continuous Oscillatory Baffled Tubes. Can. J. Chem. Eng. 2001, 79, 444–448. 10.1002/cjce.5450790318.

[ref25] Smith K. B.; Mackley M. R. An Experimental Investigation into the Scale-up of Oscillatory Flow Mixing in Baffled Tubes. Chem. Eng. Res. Des. 2006, 84, 1001–1011. 10.1205/cherd.05054.

[ref26] Levenspiel O. Chemical Reaction Engineering, 3rd ed.; John Wiley & Sons, 1998; pp 324–326.

[ref27] Phan A. N.; Harvey A.; Lavender J. Characterisation of Fluid Mixing in Novel Designs of Mesoscale Oscillatory Baffled Reactors Operating at Low Flow Rates (0.3-0.6ml/Min). Chem. Eng. Process. 2011, 50, 254–263. 10.1016/j.cep.2011.02.004.

[ref28] Parker S. A.; Amarikwa L.; Vehar K.; Orozco R.; Godfrey S.; Coffman J.; Shamlou P.; Bardliving C. L. Design of a Novel Continuous Flow Reactor for Low PH Viral Inactivation. Biotechnol. Bioeng. 2018, 115, 606–616. 10.1002/bit.26497. 29150933

[ref29] Sutherland K.; Pakzad L.; Fatehi P. Mixing Time and Scale-up Investigation of a Moving-Baffle Oscillatory Baffled Column. Chem. Eng. Technol. 2021, 44, 1403–1411. 10.1002/ceat.202000262.

[ref30] Ahmed S. M. R.; Phan A. N.; Harvey A. P. Scale-Up of Oscillatory Helical Baffled Reactors Based on Residence Time Distribution. Chem. Eng. Technol. 2017, 40, 907–914. 10.1002/ceat.201600480.

[ref31] Ahmed S. M. R.; Phan A. N.; Harvey A. P. Scale-Up of Gas-Liquid Mass Transfer in Oscillatory Multiorifice Baffled Reactors (OMBRs). Ind. Eng. Chem. Res. 2019, 58, 5929–5935. 10.1021/acs.iecr.8b04883.

[ref32] Jian H.; Ni X. A Numerical Study on the Scale-up Behaviour in Oscillatory Baffled Columns. Chem. Eng. Res. Des. 2005, 83, 1163–1170. 10.1205/cherd.03312.

[ref33] Ni X.; Pereira N. E. Parameters Affecting Fluid Dispersion in a Continuous Oscillatory Baffled Tube. AIChE J. 2000, 46, 37–45. 10.1002/aic.690460106.

[ref34] Phan A. N.; Harvey A. P. Effect of Geometrical Parameters on Fluid Mixing in Novel Mesoscale Oscillatory Helical Baffled Designs. Chem. Eng. J. 2011, 169, 339–347. 10.1016/j.cej.2011.03.026.

[ref35] Stonestreet P.; Van Der Veeken P. M. J. The Effects of Oscillatory Flow and Bulk Flow Components on Residence Time Distribution in Baffled Tube Reactors. Chem. Eng. Res. Des. 1999, 77, 671–684. 10.1205/026387699526809.

[ref36] McDonough J. R.; Murta S.; Law R.; Harvey A. P. Oscillatory Fluid Motion Unlocks Plug Flow Operation in Helical Tube Reactors at Lower Reynolds Numbers (Re ≤ 10. Chem. Eng. J. 2019, 358, 643–657. 10.1016/j.cej.2018.10.054.

[ref37] Haseidl F.; König P.; Hinrichsen O. Single-Phase Flow Residence-Time Distributions in a Rotor-Stator Spinning Disc Reactor. Chem. Eng. Technol. 2016, 39, 2435–2443. 10.1002/CEAT.201600247.

[ref38] Boskovic D.; Loebbecke S. Modelling of the Residence Time Distribution in Micromixers. Chem. Eng. J. 2008, 135, S138–S146. 10.1016/j.cej.2007.07.058.

[ref39] Bošković D.; Loebbecke S.; Gross G. A.; Koehler J. M. Residence Time Distribution Studies in Microfluidic Mixing Structures. Chem. Eng. Technol. 2011, 34, 361–370. 10.1002/ceat.201000352.

[ref40] Ni X.; Gao S. Mass Transfer Characteristics of a Pilot Pulsed Baffled Reactor. J. Chem. Technol. Biotechnol. 1996, 65, 65–71. 10.1002/(SICI)1097-4660(199601)65:1<65::AID-JCTB352>3.0.CO;2-1.

[ref41] Ni X.; Johnstone J. C.; Symes K. C.; Grey B. D.; Bennett D. C. Suspension Polymerization of Acrylamide in an Oscillatory Baffled Reactor: From Drops to Particles. AIChE J. 2001, 47, 1746–1757. 10.1002/aic.690470807.

[ref42] Avila M.; Fletcher D. F.; Poux M.; Xuereb C.; Aubin J. Mixing Performance in Continuous Oscillatory Baffled Reactors. Chem. Eng. Sci. 2020, 219, 115600 10.1016/j.ces.2020.115600.

[ref43] Oliva J. A.; Pal K.; Barton A.; Firth P.; Nagy Z. K. Experimental Investigation of the Effect of Scale-up on Mixing Efficiency in Oscillatory Flow Baffled Reactors (OFBR) Using Principal Component Based Image Analysis as a Novel Noninvasive Residence Time Distribution Measurement Approach. Chem. Eng. J. 2018, 351, 498–505. 10.1016/j.cej.2018.06.029.

[ref44] McDonough J. R.; Oates M. F.; Law R.; Harvey A. P. Micromixing in Oscillatory Baffled Flows. Chem. Eng. J. 2019, 361, 508–518. 10.1016/j.cej.2018.12.088.

[ref45] Muster-Slawitsch B.; Buchmaier J.; Brunner C.; Nidetzky B.; Gudiminchi R. K.; Harvey A. P.; Phan A. N. Oscillating Flow Bioreactors: An Enabling Technology for Sustainable Biorefining Operations?. J. Adv. Manuf. Process. 2020, 2, e10046 10.1002/amp2.10046.

[ref46] Mackley M. R.; Ni X. Experimental Fluid Dispersion Measurements in Periodic Baffled Tube Arrays. Chem. Eng. Sci. 1993, 48, 3293–3305. 10.1016/0009-2509(93)80213-A.

[ref47] Egedy A.; Oliva J. A.; Szilágyi B.; Nagy Z. K. Experimental Analysis and Compartmental Modeling of the Residence Time Distribution in DN6 and DN15 Continuous Oscillatory Baffled Crystallizer (COBC) Systems. Chem. Eng. Res. Des. 2020, 161, 322–331. 10.1016/j.cherd.2020.07.011.

[ref48] Ni X.; Stevenson C. C. On the Effect of Gap Size between Baffle Outer Diameter and Tube Inner Diameter on the Mixing Characteristics in an Oscillatory-Baffled Column. J. Chem. Technol. Biotechnol. 1999, 74, 587–593. 10.1002/(SICI)1097-4660(199906)74:6<587::AID-JCTB87>3.0.CO;2-C.

[ref49] Ni X.; Gough P. On the Discussion of the Dimensionless Groups Governing Oscillatory Flow in a Baffled Tube. Chem. Eng. Sci. 1997, 52, 3209–3212. 10.1016/S0009-2509(97)00104-8.

[ref50] Sheridan R.; Cardona J.; Tachtatzis C.; Chen Y. C.; Cleary A.; Briggs N.; Florence A.; Atkinson R.; Michie C.; Andonovic I.; Sefcik J. Effect of Oscillatory Flow Conditions on Crystalliser Fouling Investigated through Non-Invasive Imaging. Chem. Eng. Sci. 2021, 117188 10.1016/J.CES.2021.117188.

[ref51] Reis N.; Vicente A. A.; Teixeira J. A.; Mackley M. R. Residence Times and Mixing of a Novel Continuous Oscillatory Flow Screening Reactor. Chem. Eng. Sci. 2004, 59, 4967–4974. 10.1016/j.ces.2004.09.013.

[ref52] Muñoz-Cámara J.; Crespí-Llorens D.; Solano J. P.; Vicente P. Baffled Tubes with Superimposed Oscillatory Flow: Experimental Study of the Fluid Mixing and Heat Transfer at Low Net Reynolds Numbers. Exp. Therm. Fluid Sci. 2021, 123, 110324 10.1016/j.expthermflusci.2020.110324.

[ref53] Slavnić D. S.; Živković L. V.; Bjelić A. V.; Bugarski B. M.; Nikačević N. M. Residence Time Distribution and Peclet Number Correlation for Continuous Oscillatory Flow Reactors. J. Chem. Technol. Biotechnol. 2017, 92, 2178–2188. 10.1002/jctb.5242.

[ref54] Avila M.; Fletcher D. F.; Poux M.; Xuereb C.; Aubin J. Predicting Power Consumption in Continuous Oscillatory Baffled Reactors. Chem. Eng. Sci. 2020, 212, 115310 10.1016/J.CES.2019.115310.

[ref55] Crater J. S.; Lievense J. C. Scale-up of Industrial Microbial Processes. FEMS Microbiol. Lett. 2018, 365, fny138 10.1093/femsle/fny138. PMC5995164 29860483

[ref56] NiTech Solutions Ltd. Genzyme Case Study. https://www.nitechsolutions.co.uk/market-sectors/pharmaceuticals-fine-chemicals/genzyme-case-study/ (accessed May 10, 2022).

[ref57] Poon C. Measuring the Density and Viscosity of Culture Media for Optimized Computational Fluid Dynamics Analysis of in Vitro Devices. J. Mech. Behav. Biomed. Mater. 2022, 126, 105024 10.1016/j.jmbbm.2021.105024. 34911025

[ref58] Petkov G. D.; Bratkova S. G. Viscosity of Algal Cultures and Estimation of Turbulency in Devices for the Mass Culture of Micro Algae. Algol. Stud./Arch. Hydrobiol., Suppl. Vol. 1996, 81, 99–104. 10.1127/algol_stud/81/1996/99.

[ref59] Sasongko N. A.; Noguchi R.; Ito J.; Demura M.; Ichikawa S.; Nakajima M.; Watanabe M. M. Engineering Study of a Pilot Scale Process Plant for Microalgae-Oil Production Utilizing Municipal Wastewater and Flue Gases: Fukushima Pilot Plant. Energies 2018, 11, 1693 10.3390/en11071693.

[ref60] Mazubert A.; Fletcher D. F.; Poux M.; Aubin J. Hydrodynamics and Mixing in Continuous Oscillatory Flow Reactors—Part I: Effect of Baffle Geometry. Chem. Eng. Process. 2016, 108, 78–92. 10.1016/J.CEP.2016.07.015.

[ref61] Kacker R.; Regensburg S. I.; Kramer H. J. M. Residence Time Distribution of Dispersed Liquid and Solid Phase in a Continuous Oscillatory Flow Baffled Crystallizer. Chem. Eng. J. 2017, 317, 413–423. 10.1016/j.cej.2017.02.007.

